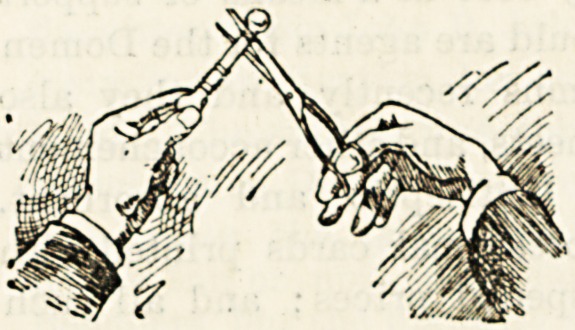# The Hospital. Nursing Section

**Published:** 1901-11-02

**Authors:** 


### The Hospital.

### Nursing Section.

Contributions for this Section of "The Hospital" should be addressed to the Editor, "The Hospital" Nursing Section, 28 & 29 Southampton Street, Strand, London, W.C.

No. 788.—Vol. XXXI. *SATURDAY, NOVEMBER* 2, 1901.

### Notes on News from the Nursing World.

### NURSES FOR THE ORANGE RIVER COLONY.

The Secretary of State for the Colonies informs us that the following forty nurses either sailed or are sailing in the *Britannic* to the Orange River Colony Concentration Camps :—Miss Gill (Matron), Miss Janet McFarlane Livingston, Miss Catherine Elizabeth Macvicar, Miss Susan McLean McVean, Mrs. Eliza Rintoul, Miss K. G. Jones, Miss Isabella Frederica Bain, Mrs. Mary Elizabeth Lindsay, Miss Annie Thompson, Miss Groundwater, Miss A. A. Partridge (Matron), Miss H. Batchelow, Miss N. Blew, Miss A. F. A. Brown, Miss E. Clarke, Miss G. E. Custance, Miss G. Cooper, Miss F. C. Davis, Miss E. M. Denne, Miss E. F. Fearis, Miss C. W. Jones, Miss E. E. Kirkpatrick Picard, Miss I. Maxwell, Miss I. May, Mrs. M. Miller, Miss L. J. Mount, Mrs. E. Mumford, Miss F. R. Russell, Miss L. A. Tompkins, Miss A. Warren, Miss MacCowan, Miss J. P. Macintyre, Miss F. M. Golding, Miss E. R. Terry, Miss L. Watchorn, Miss E. T. Kristianson, Miss I. M. Wishart, Miss E. Blanc, Miss J. D. MacGregor, and Miss J. B. Reid. Most of these nurses were, we believe, selected, at the instance of Mr. Chamberlain, by the matrons of well-known hospitals or infirmaries. Mrs. Mary E. Lindsay was trained at Edinburgh City Hospital and the Royal Infirmary, Dundee. Miss F. C. Davis was trained at the Southwark Infirmary, and has since been staff nurse at the Miller Hospital, Greenwich. Miss E. F. Fearis was trained at the Victoria Hospital, Folkestone, and has since been attached to the private nursing staff of the Royal United Hospital, Bath. Miss C. W. Jones was trained at the Southwark Infirmary, where she was afterwards sister, and has since been sister at the Wirral Hospital, Liverpool, and the City Hospital, Liverpool. Miss I. Maxwell was trained at the Deaconesses' Institution and Hospital, Tottenham ; she has since been sister at the Mission Hospital for Jews at Jerusalem, and has also been attached to the English Nursing Institute at Mentone. Miss F. R. Russell was trained at the Southwark Hospital, and has since been staff nurse at the Miller Hospital, Greenwich, and night superintendent at the Hahnemann Hospital, Liverpool. The conditions on which the appointments have been made are : free passage out and back, rations, salary £10 per month, and a sum of £10 for uniform. It will be observed with satisfaction that there are two matrons in charge of the detachment.

### NURSES IN THE CONCENTRATION CAMP AT MIDDLEBURG.

The Medical Officer at Middleburg testifies in a striking manner to the value of the work of nursing sisters in the Burgher Camp. He says that every day "our sisters" teach the Boer women something new, and every month convert several scores of irresponsible and careless women into better methods. Each of the eight nurses under him he describes as "practically the mother of hundreds of people of all ages," and he continues : "I have often seen our sisters feel inclined to put their hands to their heads and run away anywhere, but a moment more and they are forgetting themselves and their feelings in useful work. They never did more useful, more blessed work, in any hospital in their lives, and though few but the doctors and those in charge of these camps appreciate the value of their work, and very, very few feel a grain of gratitude for it, they will, most of them, live to see the fruits of it, I feel sure." As to the work, he says : "This month of July has been the most harrowing time for the nursing sisters and myself that we have ever seen, or shall ever see, in our lives. With every one of the staff working like niggers, often day and night, with abundance of all that was necessary, our death rate rose to 413 last month, or just about fifty per thousand."

### THE WAR NURSES.

The *Dunera* arrived at Southampton from South Africa on the 24th inst. with the following nursing sisters on ship's staff : — A.N.S. Superintendent Sister M. G. Hill (rejoins ship) ; K. Moxon (rejoins ship) ; H. Miller (wishes to be employed on home station or will return to South Africa) ; H. Kenealey (rejoins ship) ; E. Kitching (disembarked at Plymouth) ; E. K. Sharpe (resigns) ; A. McLeod (requires one month's leave and returns to South Africa) ; G. C. Moxon (wants home station employment). Nursing Sister R. Lawless was invalided home.

### A MEMORY OF THE CRIMEA.

A painful incident which has befallen a West End church recalls in a curious way the memory of Eliza Polidori, one of the band of devoted women who worked under Miss Florence Nightingale in the Crimea. Eliza Polidori was the sister of the young physician of that name who acted for a time as Byron's private secretary, and whose restless career ended tragically at the age of 26. Her sister Lavinia became the mother of the Rossettis. It was comparatively late in life when she herself responded to the call of the Red Cross. For her distinguished services in the cause of the sick and wounded Allies Miss Polidori received from the Sultan of Turkey a magnificent cross set with jewels. The gift eventually came into the hands of the recipient's niece, the late Christina Rossetti, who presented it to Christ Church, Woburn Square, as an altar cross. It is this beautiful ornament, of so much historic as well as artistic interest, which some miscreant has sacrilegiously stolen. It is supposed that he remained concealed in the church at the end of the Sunday evening service.

### OUR CLOTHING DISTRIBUTION.

We have been favoured with several communications from our readers who are interesting themselves in our distribution of clothing at Christmas, and as some of them desire to know whether it is "too early" to send in contributions, we think it advisable to state that December 16th is only named as the latest date. If any of our friends have their parcels ready, and would like to forward them at once, we are perfectly willing to receive them. There is ample room for storing in the offices of The Hospital, and we shall rejoice if the accumulation of gifts becomes quite unwieldy before the end of November. Each parcel should be addressed to the Editor, 28 & 29 Southampton Street, Strand, London, W.C., and should be marked "Clothing Distribution."

### THE SUSPENSION OF A SUPERINTENDENT NURSE.

Full inquiry will, we hope, be made into the case of Miss Newbury, the superintendent nurse at Dudley Workhouse Infirmary. The discussion of the matter by the Board of Guardians was far from satisfactory. It appears that, though Miss Newbury has been suspended, she is not aware of the nature of the charges preferred against her, and she herself makes complaints against three nurses, and alleges that the master and matron of the workhouse have hampered her in the discharge of her duties. The Local Government Board, to whom an appeal was addressed by her, have been informed in reply that the Dudley Guardians "believe that the retention of Nurse Newbury would be prejudicial to discipline amongst the nursing staff, and that therefore they request approval of her suspension and removal from office." But it is not explained why the guardians have arrived at such a conclusion. Neither are they unanimous. Mrs. Powell warmly defends the superintendent nurse, and affirms that the guardians would act more wisely if they removed the only male nurse on the staff. Something has been said about Miss Newbury "defying" her superior officers, but this and other points the Local Government Board will doubtless investigate before they comply with the demand of the guardians.

### YORKSHIRE GRATITUDE.

A year ago we noticed the inauguration of a District Nursing Association at Richmond, Yorkshire, and we now learn with pleasure from the first annual report and balance-sheet for the 12 months ending August 31 that the result of the experiment is highly satisfactory. The nurse paid upwards of 4,000 visits to 182 cases—a convincing proof of the need of the organisation—and the fact that it is appreciated by the residents of Richmond is shown by the existence of a balance in hand of £15. An interesting feature of the work has been the gratitude manifested to the nurse by some of the very poorest patients, who—not having, even in a country town, a garden—gathered watercress and mushrooms and sent them to her. She has also been the recipient of fruits and flowers from the more fortunate possessors of gardens. These little attentions are not the less welcome because they are not at all expected.

### "ANGELS OF MERCY" ABOARD SHIP.

The *Madras Times* referring to the articles in The Hospital on "Nurses Aboard Ship," says that the sick who go home from India are mostly of that class where sickness requires the nurse even more than the doctor. "On board ship the majority have to do without the ministrations of this white-capped, gentle-handed, clear-brained angel of mercy. A doctor the ship nearly always carries, a clever young man mostly, but a nurse, unless a patient is rich enough to pay for his own, never. A man is abandoned to the ministrations of the stewards—often valuable and well-intentioned, and cheerfully given, but always ignorant—a woman to the stewardess." Our contemporary appeals to the P. & O. Company to rise to the occasion, even on the strength of the paying "coal primage," and adds that if Sir Thomas Sutherland and his colleagues decline to respond to it "perhaps the German lines may, and reap another reward for intelligent progressiveness."

### A QUESTION OF PRINCIPLE AT BRISTOL INFIRMARY.

With reference to our remarks last week respecting the rumoured intention of the authorities of the Bristol Infirmary to grant a three years' certificate to a nurse who has not passed through the usual course of training, another correspondent, who should also be in a position to know the facts of the case, states that the nurse in question "has never been in charge of wards, except as an assistant doing probationer's work, and always working under a charge nurse." This, it is added, "she has been doing for the past two years and ten months." We also publish, in another column, a letter from the resident medical officer at Stapleton Workhouse, who states that no certificate of training has ever been promised by him or by the guardians to her.

### RESIGNATION OF THE HEAD NURSE AT CROYDON INFIRMARY.

The Croydon Board of Guardians met on Tuesday. The supplementary agenda included the following item :—" 20. Letters—Miss C. E. Finnis, head nurse at the infirmary, resigning her appointment." The matter was not discussed. During the public sitting of the Board there was no reference to the troubles at the infirmary, owing, perhaps, to the fact that no Infirmary Committee's report was before the Board.

### WORKHOUSE NURSING IN IRELAND.

The question of workhouse nursing is still actively engaging the attentions of Irish boards of guardians. A meeting of delegates from Ulster unions was held on Saturday last in Monaghan Workhouse, the object of which was to suggest means of limiting the authority of the Local Government Board, and preventing them from unduly interfering with the decisions of the local bodies. Six unions and a few of the district councils were represented. The nursing question was the only one under discussion. One gentleman, in retailing the grievances of the union he represented, said that since the Local Government Act was framed they actually had to appoint six nurses for only 254 patients, and 15 years ago, with an average of 370, they only employed three. Now, it may be asked how can six nurses—two of these we will presume are on night duty—possibly manage to take sufficient care of 254 patients. ? It certainly is not humanly possible to do it, unless the nurses of the union in question are made of more than ordinary material. A delegate from another union complained that the Local Government Board had not permitted the guardians to take in and train a probationer. He asserted that a nurse who had been trained in that particular workhouse fills at present a lucrative post in an English union infirmary, and he condemned the interference of the Local Government Board in depriving other English unions of a similar privilege. The delegates drafted a number of resolutions—one, that the present Local Government Board should be abolished; another, that there should be a court of appeal consisting of members of the County Council; and a third requesting His Majesty's Government to legislate on the matter in the next Parliamentary session. This is a pretty tall order.

### CONTINUED FRICTION AT WALSALL.

Both the medical officer and the superintendent nurse at Walsall Workhouse Infirmary find it extremely difficult to work with the master and matron. At a meeting of the Board of Guardians Mr. Lavender is reported to have observed that there was no good in denying that friction existed. He went on to say that "the officials could not do their best work when such a state of things existed. It must be ended or mended, and unless it was ended or mended the Board would either have to dismiss officials or ask for their resignations to be sent in." This is all very well, but under the present system, with a Board of Guardians who allow the master of the workhouse to dress up the domestics in nurses' uniform, it is not unlikely that if all the present officials resigned to-morrow the new staff would speedily discover that the avoidance of friction was impossible. Here, as elsewhere, the necessity for giving the superintendent nurse absolute control in the infirmary under the medical officer, is increasingly apparent.

### PROGRESS AT RADCLIFFE.

The subscribers of the Radcliffe District Sick Nursing Association have at length been able to decide to extend their work and obtain the services of a second nurse. This step is thoroughly justified by the annual report, which shows that the receipts exceeded the expenditure by £43 16s. 3d., and by the fact that the Association have now in the bank nearly £400. It is true that £100 of this sum came from the carnival, but as the amounts contributed by the workpeople and trades associations continue to increase, and as the need for a second nurse is very great, there was no sufficient reason for delaying her appointment. Mention was made at the meeting of the fact that in the early stages of the life of the Association there was a strong prejudice against the nurse entering the homes of the poor, while now she was welcomed everywhere. The experience of Radcliffe is not unusual, but it is always interesting to hear of the district nurse overcoming prejudice.

### INCORPORATED SOCIETY OF TRAINED MASSEUSES.

An interesting lecture on "Fractures" was given at the Trained Nurses' Club, Buckingham Street, Strand, on Friday last, by Mr. P. L. Barnard, M.S., F.R.C.S., assistant surgeon to the London Hospital. It was delivered at the request of the Council of the Incorporated Society of Trained Masseuses, for whose members it was especially intended, and the lecture should be of the greatest service to those who have to carry out the treatment of fractures by massage and passive movements. The admirable lecture, and the very excellent diagrams, limelight views and models by which it was illustrated and explained, were much appreciated by an enthusiastic audience of masseuses and nurses. Unfortunately, very few outsiders could be admitted; but the lecture-room is not large, and the club members and the members of the Society of Trained Masseuses had, of course, the first claim.

### A SUCCESSFUL BAZAAR.

The committee who worked so hard to secure the success of the bazaar in aid of the Haggerston and Hoxton District Nursing Association must feel amply rewarded for their labours. At a meeting held at the Nurses' Home subsequent to tea, served by the nurses, Dr. Oliver read the financial statement, which showed that after deducting the expenses incurred, the funds of the Association had benefited by the bazaar to the extent of no less than £612. It will be remembered that the Shoreditch Town Hall was gratuitously placed at the disposal of the committee for five days; but even bearing in mind this privilege, the amount is very substantial and reflects credit upon all concerned.

### THE NURSES' HOSTEL COMPANY.

The fourth annual report of the Nurses' Hostel Company, Limited, was adopted at the annual general meeting on Friday. The balance of income over expenditure during the financial year just closed is £574, and it was agreed to pay the dividend of 4 per cent. recommended by the directors, £100 being added to reserve, and a balance of £18 11s. 6d. carried to the next account. The income of the Hostel shows a slight increase over that of the previous year. With the exception of a few weeks in August and September, the home has been almost continuously full, and latterly it has been so crowded that the management has had to refuse guests.

### A NURSE FOR PWLLHELI.

It is very satisfactory to note that the people of the picturesque little town of Pwllheli have taken up with enthusiasm the movement for the establishment of a nursing association. They are following the good example of Portmadoc, Blaenau Festiniog, Dolgelley, Barmouth, and Bala. In each of these places, it appears, the nursing movement is already an assured success, financially as well as otherwise, and in some instances the balance on the right side is very substantial. At the initial meeting in Pwllheli, one of the speakers said he was sure that "once established, the nursing association would never die for want of funds," and another, who described the evening as "one of the happiest he had spent in Pwllheli," announced his readiness to subscribe 10 guineas towards the fund. An excellent representative committee was then formed. Starting in this admirable manner, the organisation promises to fulfil the brightest hopes of its founders.

### SHORT ITEMS.

The death of Miss Boys, of Lucknow, who since 1895 has been a zealous honorary worker in connection with the Zenana Bible and Medical Mission, is announced.

### Lectures to Nurses on Anatomy.

By W. Johnson Smith, F.R.C.S., Principal Medical Officer, Seamens' Hospital, Greenwich.

LECTURE III.—THE SPINE (*continued*).

A good idea having been gained of the general characters of the bones of the spine, an attempt should be made to follow carefully in a complete set of loose vertebræ the changes of form presented in different regions by each of the elements of our typical specimen. This study will soon enable us to state at once, when presented with any single vertebra, the part of the spine from which the bone has been taken.

If the bone has a short and forked spinous process (fig. 4, D); if each of the transverse processes, C, be broad and perforated by a large round hole; if the body, A, be rectangular, oblong in shape, and broad transversely, and if its upper surface presents a ridge of bone on each side; if the central or neural ring, B, be heart-shaped; if the articular processes, F, be set very obliquely; and, finally, if the whole bone, as judged by our general impression of vertebræ, be small, and light, and delicately formed, we may safely assert that it is a *cervical vertebra.*

*A dorsal or thoracic vertebra* may be distinguished by the following characters, fig. 3; a very long spinous process, D, slanting downwards, and not forked at its free end; the transverse processes, C, thick and long and directed backwards and outwards, not perforated; the body, fig. 2, A, triangular with rounded angles; the ring, B, more or less circular; the articular processes, F, fig. 3, very prominent, their smooth articulating surfaces set not obliquely, as in a cervical vertebra, but vertically. To these may be added some quite special features. On each side of the body of any dorsal vertebra between the first and the ninth bones of this row may be seen two shallow depressions or facets, H, fig. 3, a larger one extending to the upper margin of the body, and a smaller one at the lower margin. A similar depression may also be seen in front of and near the tip of each transverse process. On looking at an articulated skeleton we shall find that those three depressions on either side which in a recent bone are covered by cartilage, are resting places for ribs. The upper depression on the body of the vertebra forms with a similar depression at the lower margin of the vertebra immediately above, a single articular surface, so that the posterior extremity of each rib is attached to two vertebræ.

A *lumbar vertebra,* fig. 5, also presents well-marked characters. The spinous process, D, is not short and forked as in a cervical vertebra, nor thin and slanting as in a dorsal vertebra, but is broad and square, and set horizontally the transverse processes, fig. 6, C, are thin and long; the body, A, is oval, massive, and broad from side to side; the ring, B, is triangular. A lumbar vertebra is larger than any of the cervical and dorsal vertebræ, and its outgrowths, with the exception of the transverse processes are coarser and stronger.

Such are the common characteristics of the vertebræ in each region of the spine. As, however, the several changes in the forms of the vertebræ are developed gradually and not abruptly, it will be found as we pass along the column that the lower cervical vertebræ resemble more and more those of the dorsal region, and that as the lumbar region is approached the dorsal vertebræ lose some of their special features. Thus in the seventh and last cervical vertebra, the *vertebra prominens* as it is called, the spinous process is no longer short and forked in accordance with the cervical type, but is long and prominent and projects downwards and backwards like the spine of a dorsal vertebra. This spine can be readily felt under the skin at the back of the neck.

The arrangement of the facets on the bodies of the dorsal vertebræ for the articulation of the ribs is not constant throughout this region. The first dorsal vertebra and the last four present exceptions. The first dorsal vertebra has a complete facet for the first rib, and the usual half facet below for half the end of the second rib. The ninth vertebra has only a half-facet near the upper margin, and each of the last three vertebræ has a single complete facet.

If two contiguous vertebræ—the fifth and sixth dorsal or the third and fourth lumbar for instance—be put together in their normal relations, it will be found that in consequence of the irregular shape of the bones, and the number of processes—articular, spinous, and transverse—attached to each, all of which are bound together by ligaments, there can be but a very limited range of rotatory movement between

Fig. 4.—A Cervical Vertebra (upper view).
A Body; B Neural ring; C Transverse process; D Spinous process; F Articular process.

Fig. 5.—A Lumbar Vertebra (side view).

A Body; C Transverse process; D Spinous process.

Fig. 6.—A Lumbar Vertebra (upper view).
A Body; B Neural ring; C Transverse process.

These, with most of the illustrations to the course of lectures, have been prepared by Dr. C. W. Hogarth.

them. Such movement is less impeded in the lower cervical and the lumbar regions than in the dorsal portion of the spine; but from the third cervical vertebra to the sacrum there is really very little play between any two or three neighbouring bones. Directing our attention to the upper extremity of the spine which is directly attached to the base or lower surface of the skull, we might expect to find there some special arrangement of bones and some decided variation in form, capable of allowing that free mobility of the head by which the eyes can be turned in almost every direction. Such mobility, it will be seen in the next lecture, is permitted by remarkable differences from the ordinary form of the other cervical bones that are presented by the first and second vertebræ.

### Nursing in the American Army.

By a Sister.

The fact that nurses are henceforward a part of the United States Army goes far to prove how much their services were appreciated by that country during the Hispano-American War. Trained nurses were first introduced to the various camps under the auspices of The Daughters of the American Revolution. Very soon the improvements in the ward management and the decrease of the death rate were realised, then the people decided that female nurses were a necessity, and the petition to Congress was almost a command. Even the Surgeon-General, who was very much against women in the army and considered them "expensive luxuries," was obliged to convey telegraphic despatches to the leading cities with the request that all available nurses be sent forward at once.

### Summons to Service.

One day a brownish yellow envelope placed in my hand caused an abnormal amount of excitement, for it read—"Report to Colonel in command of 3rd Division Hospital, Camp Cuba Libre, Jacksonville, Fla." The next 24 hours was all bustle, circulating the news, paying calls and receiving congratulations, studying time-tables and attending to minor details and packing. Arrived in camp after three days' journey — through interesting and ever-changing scenery—there was plenty of work, for the first 12 nurses were preparing to go on duty and to organise the hospital, which was to prove a great tax on ingenuity and originality. Two nurses were consigned to each ward, each for 12 hours duty; owing to the intense heat, and the impossibility of sleeping during the day, it was decided that duty hours should be from midnight to midday. The nurses' tents were not pitched, so the Major secured rooms in a house close by; the beds were so strongly garrisoned by the insect tribe that rest was impossible. However, taking our straw pallets out on the verandah was a happy thought, where, 'neath the fair Southern sky and glorious moon, inhaling the sweet odours of many tropical flowers, surrounded by the orange and lemon trees, and gently fanned by the graceful waving palms, we slept, regardless of the constant presence of spiders and beetles, and of the fact that we were surrounded by coloured people renowned for theft and treachery.

### Challenging the Nurses.

Being outside the guard line, it was necessary that the stewards of the Hospital Corps should escort the nurses to and from their house, partly for protection and partly because the Corporal of the Guard and his subordinate guards dare not allow man, woman, or child to advance within the guard-line at any time and double watchfulness was necessary at night. Once we were held up by the guard, who promised to shoot if we advanced, but he did not understand that the steward's stripes were his passport in any camp at any hour. The nurses were highly amused, especially as the necessary apology was given by the Corporal and accepted by the steward with a very bad grace. Our midnight lunch for the first couple of weeks was served and consisted of hard tack and black coffee. With regard to diet, the nurses were allowed by Government "one ration in kind per day" at the rate of 25c. (one shilling), precisely the same as the enlisted men.

### No Time to Think about Discomforts.

The hospital with all that pertained thereto was enclosed in a large square and surrounded on all sides by the various regiments; within easy access of all camps. The site was seven miles from Jacksonville, though a beautiful road made the distance seem less, and very picturesque, with here and there glimpses of the snowy tents through the pines, and the river St. John could be reached in about three minutes. As the hospital square had to be cleared of all vegetation, there was very little shelter from the fierce rays of the sun, though the wards were kept cool by means of double coverings for the tents and buckets of ice. Walking was very disagreeable owing to the deep sand which was strongly heated. However, the nurses did not have time to think of discomfort for the Ambulance Corps was kept busy, and between thirty and fifty patients were admitted daily, and of course more nurses were telegraphed for.

### Scarcity of Supplies.

The commanding major being a volunteer officer, had small idea as to the requirements, consequently as the list of sickness swelled every nurse was at her wits' end for supplies, both of food and linen, and indeed everything needful was far from meeting the demand. Quite frequently even milk could not be procured, one glass per day being often the diet of a typhoid or a malaria case. When eggs were procurable, necessity demanded that whatever the consequences, they must be doled out to every patient. However, in a short time, a major from the Regulars succeeded, and the times improved. He had a dining-room built for the nurses and officers, and secured the co-operation of the Red Cross Society for table supplies. A chief steward was appointed in charge of the commissariat, who devoted all his energy to the diet question; he visited farms for miles round, with indifferent success, then telegraphed to ranch-owners in neighbouring States, and finally succeeded in having a train chartered to convey milk from Georgia in the small hours of every morning. But now there was a new difficulty; the men of the Hospital Corps were rapidly decreasing through illness owing to overwork, heat, and sulphur-water.

### A Sketch of the Work.

Perhaps a sketch of a specimen hour after coming on duty may best give some idea of the work. Getting on duty soon after noon, the nurse takes up report, and her sister retires. The diets have just been served and temperatures taken; there are perhaps a dozen sponges to be given, and all hands must help. But inquiring for Pitcher, I am informed that Pitcher was ordered to bed, as a typhoid suspect.

"Well, then, where's Tosser?"

He had to give up last night; chills, &c.

"Well, Clayton."

He is present and says: "I don't feel very well, nurse; but will help as long as I can."

The other three are perhaps perfectly sound, and we prepare for action. Suddenly a summons comes from the Major's orderly.

"Dinner is ready, nurse."

"Thank you, Swinson."

But he doesn't move. "Major says the nurses must be punctual at meals, so that the men can get through with the dish-washing."

"Bother the Major, and you too!" is the answer ready; but I remember military discipline, and meekly reply, "Very well, Swinson, tell Major I'm coming;" and with reluctance I repair to the domain of edibles. We report the short-help grievance, and it is decreed that recruits shall be selected from the camps to do ward duty for 10 days, during which time they receive extra pay, wear the brazzard, and have all the privileges attached to that magic band with its red cross.

### Training Recruits.

So next day when I get on duty I find that six brand new men who have never seen a sick room have come to assist me in caring for 76 patients, 36 of them being typhoids and the rest malaria, dysentery, and other climatic derangements. I have to learn their names and take note of the regiments they belong to, for I am required to know if one should be missing and where he is likely to be found—Mississippi, Missouri, Indiana, Carolina, Texas, and Arizona are all represented—and to save time I call a clinic, and give my pupils a theoretical and practical lesson in sponging, then presenting each with a basin, water, ice, and rags, set them to work, with the remark that I want to see which State turns out the best men. Thus, while distributing medicine it is quite possible to keep a quiet eye on all helpers. We soon realised that there was very little difficulty as a rule in getting good results from the men, for they are accustomed to implicit obedience. But just as they were trained to usefulness the 10 days expired, and the nurse was confronted with a fresh relay. Occasionally it happened that one of the band proved refractory. I remember a boy who evidently had a tiny vacuum in the cerebral cavity, for he reckoned up every cent expended on him by the War Department both directly and indirectly, and finding that the sum total showed $1 per month more than the nurse's salary, he positively refused to obey, and fully intended to do as he pleased. But a few days in the guard-room, after which he was relegated to the position of M.D. (mule driver), soon restored his better judgment.

Arrival of a Cyclone.

The idea was once entertained of building a permanent hospital in Panama Park, where Camp Cuba Libre was situated; but the arrival of a cyclone proved the place unsafe. An emergency like this brings out the soldier in his true colours and shows that chivalry is not altogether a remote dream of he past. Officers and men worked side by side without a pause for 36 hours, while the elements raged wildly for the mastery. However, owing to untiring vigilance the tent-pegs were kept in their sandy position, and when men collapsed from cold and fatigue they were swiftly carried inside the tents and their places expeditiously and silently filled, for all knew that if the fury of the storm gained the ascendancy, it meant death to many of the sick. Within the wards the doctors and nurses had not a moment's breathing space, there was a constant moving of mattresses or patients as the rain beat through every opening and saturated some of the beds. One doctor stood with bottle in hand dispensing stimulants to chilled workers on the outside, nobody could even think of food, and indeed could not get to the dining-room if they tried. The nurses had to encourage their patients and keep a constant watch for any slackening rope or swaying tent-pole, and report immediately to the corporal of the guard. During the night all available transport trains were drawn up to the hospital and the sickest patients conveyed to them for safety. But owing to wreckage and other difficulties on the line, the trains could not leave with their freight till late in the next afternoon, but the patients had good care, for hospital trains are very well fitted with supplies: there is a male nurse in charge of each section, and fully qualified doctors in charge of each train. Thus commenced the breaking up of camp and the removal of the hospital. Every week the train took fresh relays of patients to the various forts, which gave the nurses time for rest and the chance of visiting places of interest.

Founding the Army Service.

When peace was proclaimed and nurses mustered out of the service, the question of a permanent staff of army nurses was discussed; and mass meetings were held for that purpose. A bill was drawn up for the House of Representatives and Congress which finally passed with the result that there are about 200 trained female nurses now employed by Government.

Four large general hospitals are also maintained in the States. The largest is at Presidio, Cal.; a second is at Washington Barracks; a third at Fort Bayard, N.M.; and the most perfectly equipped is at Hot Springs, Arkansas, though to this trained nurses have not yet been drafted. It has accommodation for 150 patients, and about 400 are treated annually. It is a rambling construction of brick with stone trimmings; towers and gables, trophy guns and a growth of ivy on the walls give the exterior a picturesque appearance. The interior has hard-wood floors and painted walls, tile, metal and cement being used wherever available. Besides the administration building and the officers' rooms there are four wards, each about 127 feet long, 28 feet wide, and 27 feet high. It is the only hospital in the country occupying a position near medicinal waters, is heated in winter by steam, and the piazzas are enclosed in glass. With a smoking-room, sun parlour, palms and plants, good food and nursing, it is surely a pleasant change for the soldier who has been amusing himself trying to keep out of touch with Krag-Jorgensons and Mausers in Luzon or other such places.

### A Visit to an Indian Nursing Home.

By a Correspondent.

The last words that Sir Mancherjee Bhownaggree, M.P., said to me as he saw me off at Victoria Station were "Be sure you go and see my nursing home at Bombay." I arrived at Bombay at mid-day and had to leave the same evening for a twenty-four hours' journey, but I did not forget Sir Mancherjee's injunction, and one of the visits which I paid during my all too short afternoon was to the Jamsetjee Jejubhoy Hospital and the Bhownaggree Nursing Home, and I at once thought of the readers of The Hospital and the keen interest they always take in the varied scope of their professional work, so I sat down the same evening to write for them a brief word-picture of my visit.

The Home.

The Maharajah of the State I had come to visit had been good enough to put at my disposal one of his sepoys to take charge of me, so that I had no difficulty with cabs, but drove rapidly through the crowded streets with my scarlet-turbaned, blue-bloused sepoy sitting beside the driver in front. Suddenly turning out of a crowded thoroughfare, through a great gateway, we found ourselves in a delightful drive with almond trees and date palms, and cocoa-nut palms, and many another tree and shrub on either hand, and with numbers of parrots flying about everywhere, until we pulled up at a bungalow half hidden in foliage. There was no bell, so we stood for a few minutes in the porch and read the inscription:—"Arabai Bhownaggree Home for Nurses, built from a joint fund set apart by Government and by Mancherjee Meherwajee Bhownaggree, C.I.E., in memory of his sister, Bai Arabai, who died on the 23rd of November, 1888, aged 19. This home was opened by Lady Harris and cost 30,605 rupees." We looked through the wide open doorway into a spacious hall, replete with arm-chairs and comfortable lounges and dainty little drawing-room nick-nacks which showed the presence of English ladies of taste and culture. Behind this I could see another wide passage with a couple of "bikes," looking very familiar and home-like. As no one was there we strolled in, and my sepoy noiselessly stole forward with his bare feet down the passage and up a staircase holding my card in his hand as a credential as well as a demand, for the first person he should meet.

THE TRAINING.

As noiselessly as he had disappeared so he returned and led me across the shrubbery to another bungalow, and there, in a restful little room with an open door at each end and open lattice windows all down one side, sat a gentle-faced sister at her desk writing. Dressed in the "Sister of Mercy" garb of the All Saints, Margaret Street, Sisters, she looked a cool spot in a hot world. Under her kindly care I learned the methods of administration and visited the hospitals at which the nurses were trained. There are three hospitals built almost adjoining each other: The Jamsetjee Jejubhoy Hospital for general medical and surgical cases; the Bai Motlibai Hospital for midwifery cases only, and the Sir D. M. Petit Hospital for gynæcological cases only, all for natives. There is a medical school attached, and this is recognised by the Bombay University for training for the medical diploma. The nursing of these hospitals is done in part by the nursing home nurses, and in part by native men. The course is for two years, of which the chief part is spent in the general hospital, but nurses over the age of 23 may spend part of their second year in the Motlibai Hospital, and after witnessing 25 labours, and then personally conducting 25 more, they may pass their examination for a special certificate in midwifery, in addition to their general certificate in nursing.

"Are your nurses English?" I asked.

"Yes, our ward sisters are," replied my guide, "but not our probationers. That is to say, we do not train English girls, because we never get any applications. We bring out from England a trained nurse for each ward, and then they nurse the ward with Eurasian (*i.e.*, one who has some European and some native blood) and native probationers."

"Do you never promote any of your pros. to be staff nurses then?" I asked.

"Eurasians, yes; but natives never," she replied, "because native women hold such a post of subordination that they would have no authority whatever in male wards."

"How many beds have you?"

"We have 300 in the general hospital, but we do not nurse all the wards for want of funds."

NATIVE MALE NURSES.

"What is the system there, then?"

"Oh, a very simple one, practically a system of native male nurses. The young fellows go round with the native House-Surgeon, and he shows them what he wants doing, and the head 'ward boy,' who is as good as a staff nurse for practical work, gives them instructions."

"Then this is really a male as well as a female nursing school?"

"Yes; but with this difference, that the male nurses get no certificate, have no definite length of service, and are non-resident."

"Twenty-five to thirty nurses and pros. seems rather a small staff for all your work?"

"That is true; but you must remember that we do not do nearly as much for native patients as nurses do at home. In the first place the food is served, as well as prepared, by servants of their own respective castes, and these servants all live in a range of huts in the grounds close by. In the second place, unless patients are really helpless, they prefer to wash themselves. In the third place we do not give bed pans, or remove sputa or excreta, or our position would be quite lowered, according to their ideas. Of course, there are exceptional cases where we do all these things, but they are very much less done than in England."

FOOD RESTRICTIONS.

"Now, sister, I want to ask one more question before I go. Do you not find all these curious food restrictions a great nuisance?"

"Not at all; I think them quite beautiful. I am not a vegetarian, myself, but I quite understand how high-caste men and women who eat no dead bodies and touch no dead thing find it repulsive to have their food handled by people whose hands and mouths have touched of both. We do not often have very high-caste patients in, however," she continued; "but the other day we had one of exceptionally high caste, and as I was going down the ward I saw that he was eating, and I went up to see if he was quite comfortable and had all he needed; but he waived me away in a most lordly way, for had even my shadow fallen on his food I should have polluted it and he would have had to throw it away!"

I went through the surgical and medical wards and found them charmingly cool and airy, but how the souls of the home-trained nurses must squirm when they first come out and find beds with no quilts to tuck in! with no lockers in which to put things out of sight! and with patients lying huddled up outside their beds, practically naked and perspiring even then! The carriage was waiting and I had many calls to make, so that I had only time to give a good-bye pat to half a dozen little nude children who were playing about the verandah; to peep into the bare-looking theatre with its two tables which are used simultaneously; to take in the fact that nurses dressed promiscuously so long as they had a cap and apron; to realise that all the servants were men and boys; and that in the grounds the grass was rank, crows and jackdaws and flies were plentiful, and some of the drains were surface-water gutters. Sister was very happy and liked the life and the climate, which, she said, suited her perfectly; but then she had one of those saintly faces which would sweetly smile from out of a purgatorial flame if she were only conscious that she were helping some poor pain-stricken body to rest, or a sin-stained soul to paradise.

### Presentations.

SOUTHWARK INFIRMARY.—Miss E. M. Byles, who is shortly leaving to take up the duties of matron at Lambeth Infirmary, has been presented with a silver toilet set and two framed engravings from the medical staff, matron, steward, chaplain, and nursing staff; also a silver inkstand and oak Chippendale tray from the domestic staff, scrubbers, and laundry staff, as a mark of their esteem, and appreciation of her untiring efforts to promote the welfare of all in the institution.

ECCLESALL BIERLOW UNION.—An interesting ceremony took place last Thursday evening at the hospital of the Ecclesall Bierlow Union, when a presentation on behalf of the Union staff of a silver tea tray, and of the hospital staff of a silver teapot, cream-jug, and sugar basin, was made to Mrs. Lipscombe, the superintendent nurse, by Mr. Thomas Smith, clerk to the guardians. In making the presentation Mr. Smith referred at some length to the good work done by Mrs. Lipscombe during her seven years' service at Ecclesall, and the regret which every official felt on learning that she had been compelled for a time to relinquish her duties. During the seven years Mrs. Lipscombe had gained the love of all her patients by her kindness, sympathy, and attention, and by her courtesy and zeal the respect of every officer with whom she had come in contact. Dr. Gale, Medical Officer of the Workhouse, reminded his audience of the great changes in administration which had taken place during Mrs. Lipscombe's residence with them. No one could regret her departure more deeply than he did. He hoped that the small presentation would in some slight way remind her of the esteem in which she was held by everyone at Ecclesall. Mr. Tarrant, the master, made some appropriate remarks, assuring the retiring superintendent nurse that her absence would be as deeply felt by the Union officials as by the hospital staff. Mr. Kennedy, the chaplain, also spoke to the same effect. The same evening Nurse Griffiths was presented with a travelling-case and a silver inkstand from the various officials, and much regret was expressed that she had decided to relinquish her appointment and join Mrs. Lipscombe in a well-earned rest.

### Mr. Chamberlain on Nursing.

THE SCOTTISH BRANCH OF THE COLONIAL NURSING ASSOCIATION.

On Tuesday afternoon the Colonial Secretary and Mrs. Chamberlain were present at a meeting in Edinburgh held for the purpose of creating interest throughout Scotland in the work of the recently-founded Scottish branch of the Colonial Nursing Association, and raising a sum of £1,000 towards the invested funds. The Lord Provost of Edinburgh presided.

Lord Balfour moved the principal resolution, which commended the objects of the Association to the people of Scotland. He said that there could be no more laudable object, and he did not think double the sum asked for was too large to expect.

The Lord Provost of Glasgow, Mr. Cochrane, M.P., and others also spoke in similar terms.

Mr. Chamberlain, who was received with loud applause, in rising to acknowledge a vote of thanks to Lady Balfour and Mrs. Chamberlain, said :—My Lord Provost, ladies and gentlemen, I obey your call, Sir, although I feel that I am in the position of an intruder, or, at least, of an " extra " to this meeting. I was afraid that my engagements would have prevented me from attending, and accordingly I was, perhaps, glad to see that my name was not down on the programme. But as it happens that I am in Edinburgh at the time, I can only say I feel it a privilege to have been permitted to come here, and it is with the very greatest pleasure that, on behalf of my wife, who has taken, as you know, the greatest possible interest in this Association, I tender her thanks, in the first place, to Lady Balfour of Burleigh, who has kindly placed her great influence and popularity at the service of the Association; to those who have so generously contributed to the funds already, and to those who in the future are, no doubt, going to do so. I should find it difficult to add much to what has been already said as to the objects and the necessity of such an Association.

### The Standpoint of the Secretary of State.

Colonel Marr-Stuart has spoken of the services of the nurses to our soldiers abroad, and the Lord Provost of Glasgow spoke of their services to their own countrymen, whatever their capacity might be. I approach the subject, perhaps, from the official standpoint—from the point of view of the Secretary of State, who is charged with the administration, or, at all events, with the responsibility for the administration of a great number of colonies existing under very various circumstances in all parts of the world. We hear, nowadays, a great deal about Imperialism, and sometimes that word is used in a controversial sense. But there is one sense in which I am perfectly certain all of us, whatever our political opinions may be, are true Imperialists. There is no man belonging to the United Kingdom who can fail to be proud of what his countrymen have done in the way of civilisation, law, order, justice, and religion to the distant corners of the earth. In that work, no doubt, this country of Scotland has borne a large part. The Lord Provost of Glasgow, in his modesty, has said little enough upon that subject. I can say more, for in my experience I find that in every branch of the great service which administers these colonies Scotsmen are conspicuous, and the ground for our pride lies in this—that we are proved to be a great governing nation by the acts of our children and our fellow-subjects abroad. No matter what the difficulty may be, there are always to be found in England, or Scotland, or Ireland the men—often very young—who are qualified to undertake it. No other country has hitherto been equally fortunate. I have seen the work of empire going on in Egypt, for instance. I am aware of the details of the work, every day of my life, in the numerous colonies for which, as I have said, I am responsible. The complexity of the administration, the extraordinary novelty, and the difficulty of the work are, I think, sometimes hardly appreciated, and when you think that all this vast machinery works smoothly, on the whole, to the great credit of the country and the enormous addition to its prosperity and influence, surely there is no one of us but who is proud of the men who enable us to make this boast.

### An Obligation of Empire.

But if it be true that our debt is so great, let us not forget the obligation. Mrs. Chamberlain, in the article which she wrote some time ago describing the work of this Association, appropriately called it an obligation of empire. We remain at home. These men who are sustaining the honour and the interests of the Empire abroad have difficulties to encounter which we can hardly, perhaps, sufficiently appreciate. But there remains something for us to do. There remains for us to support them in their work, to make allowance even if occasionally, under circumstances that would try a statesman of the highest rank, they make mistakes; to defend them when they are attacked, and especially when they are attacked by their own countrymen. That is our duty; but beyond that we have to do everything we can to make their lives more endurable, and to preserve lives which are, above all others, valuable to the Empire. What are the conditions of life? I am not speaking now of those great self-governing colonies to which our assistance is not needed, and in which life is as agreeable as it is here in the mother country. But think of the vast dominion over which the British flag flies, which is not a natural home for white men—for Europeans—in which the climate is a constant source of danger. If we are to continue this great work with effect and success, we must do everything in our power to make life more healthy where now it is, unfortunately, so dangerous. A great deal is being done in many ways. I would refer to those new schools of medicine in London and in Liverpool which have been established especially for the investigation of tropical diseases. The discoveries which they have already made are most promising, and I venture to look forward to a time, which I shall not, perhaps, live to see, in which many of those colonies now reputed unhealthy may be made, at all events, as healthy as India or some other places which have long been settled and civilised. They also, in the earlier days, were considered to be the white man's grave; but that is no longer the case, and I think we may be hopeful and sanguine in our work, and may be able to extend the improvements thus made to the other colonies. Meanwhile, think for a moment what is the condition of a man employed by the Government in this great and important work in a colony to which his constitution is not adapted, where circumstances are not thoroughly understood, struck down by an illness which might not be fatal, but which will inevitably be fatal if it is not properly watched. Think of that man with no society, no friends, no relatives to look after him, and, as has been the case in the past, with no woman of his own colour, of his own race—no woman with her kindly sympathy to attend upon him. This is what this Association undertakes, so far as the means are placed in its power to obviate. I believe the work is a Christian work, a charitable work, and an Imperial work, and I heartily recommend it to you.

### The Royal British Nurses' Association.

GENERAL COUNCIL MEETING.

There was not a great deal of business for the meeting of the General Council of the Royal British Nurses' Association on Friday afternoon, a sure sign, Mr. Fardon thought, that matters were going on satisfactorily. Mr. Pickering Pick was in the chair, and there was a fair attendance. A letter regretting her inability to be present had been received from the President, H.R.H. Princess Christian.

The following report of the Executive Committee was adopted unanimously:—

The Executive Committee have to report that since the last meeting of the Council in June 45 nurses have been registered, 45 new members have been elected, one has resigned her membership, and three deaths have been reported. The commttttee report with much pleasure the continued satisfactory progress of the fund for erecting a Settlement for aged nurse members of the Corporation. The fund is much indebted to Miss Armstrong and Miss Busby, through whose kind exertions a successful sale was held at Ventnor in August, and the sum of £70 realised for the Settlement. The committee desire to express their cordial thanks to Lord and Lady Brassey for their kindness in promising to lend their house in Park Lane for the sale, which has been fixed to take place in February, on behalf of the Settlement. The annual Conversazione has been fixed to take place at the Town Hall, Kensington, on Tuesday, December 3rd. The committee are glad to announce that Dr. Jane Walker has very kindly consented to deliver the first Sessional Lecture to members on the subject of Tuberculosis on the preceding day. The following ladies have kindly consented to act as lady consuls in their respective localities: Mrs. Turner, Chaseley, Rochdale; Miss Ronaldson, matron, Salisbury Hospital, Rhodesia. Financial report, July, August, and September, 1901: receipts, £247 9s. 10d.; expenditure, £259 9s. 4d.; bank balance, September 30th, £79 18s. 11d. Royal British Nurses' Settlement: invested in trust stocks, £500; in general account at banker's, £129 0s. 4d.; total, £629 0s. 4d.

The Hon. Treasurer, Mr. Langton, said that the R.B.N.A. Settlement scheme was a matter which must claim the interest of all supporters of the Association, because it enabled the Association to deal with the old age and sickness of its members, and thus to benefit a large number. He was not prepared to say what amount of money would be needed, but he did not think he should sanction the expenditure of any money until the possibility of beginning to build was within sight. Not only would the building be more expensive than at first appeared, but there would be the furnishing also to think of, and he was afraid to say how many pounds would be wanted for that purpose.

Miss Thorold thanked Mr. Langton for speaking plainly on a matter which she felt sure caused some anxiety to the members; the Association ought not to rush into building until there was sufficient money in hand to pay for it.

The Hon. Treasurer, continuing, said he had recently been shown, by a matron, a very expensive aseptic instrument cupboard; but there was no money to buy the instruments. It would not do to act on such lines with regard to the Settlement.

Several letters were read by the Hon. Secretary, Mr. Fardon, from Miss Georgina Macvitie, on a subject discussed at the last Council Meeting, viz., the explanatory footnote added to the roll of midwives. The subject, as Mr. Fardon explained, was now a matter of ancient history, but in accordance with the expressed wish of the writer he had read the letters.

There was some discussion on the words "diploma" and "certificate," after which it was moved by Miss Thorold that a reply be sent to the writer, pointing out that it was decided to insert the footnote rather than go to the expense of altering the word in the case of each name in the roll.

Mrs. Dacre Craven thought that the writer should be thanked for her criticisms and remarks; and the Secretary was instructed to write a reply in accordance with these suggestions.

There being no further business the meeting terminated with a vote of thanks to the Chairman.

### Novelties for Nurses.

By Our Shopping Correspondent.

GARMENTS FOR ILLNESS.

Miss M. Middlemas has patented a convenient arrangement for the clothing of bedridden or helpless patients. The inconvenience of the usual methods of fastening clothing is felt every time the garments of a helpless patient have to be changed. Great advantage will be experienced by allowing Miss Middlemas, of Rennington, Alnwick, to supply suitably made garments in cases where little disturbance of the patient as possible is essential. She can adapt her method to the size and style required.

A NOVEL SCISSORS-SHARPENER.

The illustration shows better than any words can describe the simple little contrivance which I desire to introduce to the notice of my readers. This little glass bar really has the properties claimed for it; and in saying this I am giving it the best recommendation possible. Every woman knows the hindrance and annoyance caused by a blunt pair of scissors. Hitherto she has had to relinquish this article of constant use whilst the process of sharpening took place. Now for the cost of this sharpening she can procure this simple little addition to her workbox, and, whenever necessary, can bring her scissors into working order again. The agents are Messrs. Ellen & Co., 118 Fenchurch Street, who will send the sharpener post free for fourpence. But the simpler way is to order it from a draper.

WARM WINTER GARMENTS.

The clumsy flannel garments worn by a past generation have had their day. Soft, well-shaped elastic underclothing has now nearly entirely taken their place. At first the public complained that these garments, so well-fitting at first, speedily became unwearable by shrinkage in the laundry. Manufacturers are meeting this objection, and the Knitted Corset and Clothing Company, of Nottingham, produces unshrinkable and beautifully made underclothing of all descriptions. It is economy in the end to use only the best for garments in constant wear. Now that the weather is both cold and changeable I advise my readers to write to Nottingham for a catalogue of all the productions of the Company.

CLOAKS AND CAPS AT GARROULD'S.

Almost everything a nurse can possibly require, either for her patients or her personal outfit, can now be obtained at Messrs. Garrould's. Each department stocks something that sooner or later a nurse is sure to want; and invalid chairs and sofas, travelling trunks, indiarubber goods and baths, are some of the larger things I noticed when I called a day or two ago. Cloaks, I was told, were mostly in demand, for the summer has gone and autumn rains and winter cold have now to be faced. The "Canterbury" is an improved cycling cloak in winter cheviot serge or all-wool army cloth; the lower part is supported by shoulder-straps hidden by the circular cape. For riding in a high wind, however, I should recommend the "Wellesley," consisting of a skirt and three-quarter length coat; a coat is warmer than a cape, besides being less liable to flap uncomfortably. This can be had in all-wool melton cloth or cheviot winter serge, and is a very neat and suitable costume, quite in keeping with the plain uniform bonnet. It is made in black navy blue, and grey, and is little more expensive than the cloak. All the cloaks are made of materials which are "guaranteed all wool and thoroughly shrunk," a most important point to remember when you are buying a cloak that has to withstand all weathers. The "Honora" is one I specially liked; it is a plain circular cloak falling straight from the collar and buttoning down the front; there is no cape nor frill, and it has a very neat appearance. Bonnets and caps are also very varied, and among the latter I noticed quite a new one—the "Pretoria" cap, made somewhat after the pattern of a Dutch *kapje*, but not so large. It is made of fine lawn, edged with embroidery. "Belmont" is another name of South African significance; this is a pretty cap of fine spotted muslin, with a frilled edge. "The Army Cap," as its name implies, is of the pattern worn by the Army Sisters, folded round the head and falling down over the neck; it is made of fine washing cambric or book-muslin. The ever-popular "Sister Dora" cap is quite one of the prettiest patterns, I always think; but there are many others, to suit a variety of tastes. In the boot and shoe department, nurses who suffer from "flat-foot" should inspect the patent instep-arch rest, for wearing inside the ordinary boot as a means of support to weak ankles. Messrs. Garrould are agents for the Domen belts, described in these columns recently, and they also stock Hartmann's wood-wool sheets, and other accouchement necessaries, which are both antiseptic and absorbent. Nurses can now have their professional cards printed with any wording they require, at special prices; and all such things as charts, clinical thermometers, and the surgical instruments required by nurses, are supplied—and, in fact, my readers cannot do better than send for the new edition of the "Red Cross Catalogue," and keep it by them as a book to refer to when any nursing requisite is wanted.

### Appointments.

Ballymena Workhouse Infirmary.—Miss Emily Bloxham Smyth has been appointed superintendent nurse. She was trained at the nurses' home and training school in connection with the Royal Hospital, Belfast.

Barton-upon-Irwell Union Infirmary.—Miss Ada Courtney has been appointed charge nurse. She was trained at Chorlton Union Infirmary.

Basford Infectious Hospital.—Miss Mary Emerton has been appointed nurse. She was trained at the General Infirmary, Northampton, and has since been charge nurse at Saffron Walden Hospital, and sister at Bagthorpe Hospital, Nottingham.

Bethnal Green Infirmary.—Miss M. A. Bond has been appointed sister. She was trained at Leeds Union Infirmary.

Bolton Borough Hospital.—Miss Helen A. Amos has been appointed matron. She was trained at the General Infirmary, Northampton, and has since been matron at the Stratford-on-Avon Joint Hospital, charge nurse at Plaistow Hospital, and charge nurse under the Metropolitan Asylums Board.

Bolton Infirmary.—Miss Margaret Frances Malvany has been appointed matron. She was trained at the Infirmary for Children, Liverpool, and the Royal Infirmary, Liverpool, and has since been senior sister at the Royal Albert Edward Infirmary, Wigan, and for the last three years matron's assistant at the Mill Road Infirmary, Liverpool.

Bomba, British Central Africa.—Miss G. G. Micklethwait and Miss M. M. Beales have been appointed by the Crown Agents for the Colonies nursing sisters at Bomba, British Central Africa. They were both trained at Guy's and Shadwell Children's Hospitals.

Cardiff Infirmary.—Miss K. Dora Mackenzie has been appointed sister of the women's medical and surgical wards. She was trained at Chichester General Hospital for three years, and has since been sister at St. Bartholomew's Hospital, Rochester, and sister at the Children's Infirmary, Myrtle Street, Liverpool.

Caterham Asylum.—Miss Mira Ellen Pickett has been appointed superintendent nurse. She was trained at Southwark Infirmary, and has since been head nurse at Kensington Infirmary, and charge nurse at Brook Fever Hospital, London.

Davos Invalids' Home, Davos Dorf, Switzerland.—Miss H. Atchill has been elected matron. She was trained at the Middlesex Hospital, and also at the Rotunda Hospital Dublin. She has since held sister's posts in the Staffordshire General Infirmary and the Monsall Fever Hospital, Manchester. For the last four and a half years she has been matron of Dover Hospital.

Dundee Parochial Hospital.—Miss Margaret M. Purvis was appointed charge nurse. She was trained at Burnley Union Infirmary for three years, and has since been district nurse at Bolton.

Hungerford Union.—Miss Sarah A. Middleton has been appointed assistant nurse. She was trained by the Meath Workhouse Nursing Association at the Invalid Asylum, Stoke Newington.

Leavesden Asylum, Herts.—Miss Rose Cumming has been appointed superintendent nurse. She was trained at the National Hospital, Queen Square, and has been charge nurse at the Fulbourn Asylum, Cambridge; and midwife at the Maternity Charity and Nurses' Home, Plaistow.

Market Harborough Union Infirmary.—Miss Grace M. Girdler has been appointed nurse. She was trained at Northampton Infirmary, and has since been staff nurse at the Fountain Fever Hospital, Tooting, staff nurse at the Glasgow Fever Hospital, and has just returned from nursing sick and wounded soldiers in South Africa.

Monsall Fever Hospital.—Miss Emily Taggart has been appointed sister. She was trained at Leeds Union Infirmary.

St. Leonard's Infirmary, Shoreditch.—Miss Ethel J. Atkins has been appointed matron. She was trained for three years at St. Bartholomew's Hospital, London. She has since been sister at the City Hospital, Birmingham; assistant matron at the Western Hospital, Fulham; matron at the Cuddington Hospital, Surrey; and matron at the Park Hospital, Hither Green. During 1900 and 1901 Miss Atkins was nursing in South Africa, and has been acting superintendent sister of the hospital ships, *Lismore Castle* and *Dunera*. Miss M. E. Brentnall has been appointed ward sister. She was trained at Chorlton Union Hospital, and has since been staff nurse at Islington and Hinckley Union Infirmaries.

Sevenoaks Workhouse Infirmary.—Miss Mary King has been appointed superintendent nurse. She was trained at Sculcoates Union Infirmary where she has since been charge nurse.

Stirling District Asylum, Larbert. — Miss Alice McLaren and Miss Emilie Foskett have been appointed assistant matrons. Miss McLaren was trained at Glasgow Royal Infirmary, and has since been night superintendent at the Birmingham Infirmary. Miss Foskett was trained at the East Sussex Hospital, Hastings, and has since been assistant matron at the Refuge Home, Belfast, and ward sister at the Union Infirmary, Beckett Street, Leeds.

The English Nurses' Institute, San Remo.—Mrs. Lucie Marsh has been appointed nurse. She was trained at the Birkenhead Borough Hospital; she has since been staff nurse at the St. Marylebone Infirmary, London, and has done private nursing at Bromley, Kent.

Wakefield Infirmary.—Miss Hylda M. Barnacle has been appointed sister. She was trained for three years at the Union Infirmary, Leeds.

### Echoes from the Outside World.

### AN OPEN LETTER TO A HOSPITAL NURSE.

Now that the King and Queen have returned to London, both looking extremely well, fresh life is infused into all the doings at the West End of the metropolis. The King himself is taking an immense amount of interest in the alterations which are being carried out at Buckingham Palace, and just now spends a good deal of his time there. But, like his mother, he evidently looks upon his castle in Berkshire as the nearest approach to "home" which a Royal personage is allowed to possess, for it is to Windsor that he has ordered the celebrated picture of Queen Victoria by M. Benjamin Constant to be sent. It will be remembered that the picture was accorded the place of honour at the Royal Academy during the past summer, and now that it is the King's property he has arranged for it to be hung in the dining-room at Windsor where at present there is only one other picture on the walls. The lions and zebras which have just arrived in London from Calcutta as a present to Edward VII. from the Emperor Menelik will, it is almost needless to say, not be sent to Windsor, and probably the nation, through the Zoological Gardens, will benefit, more than the King individually, from this strange gift.

This week will be always marked with red letters in the Royal Calendar, for Saturday will witness the return of the Heir Apparent and his wife from their Colonial tour. On Friday the travellers will reach Portsmouth, having been met by the King and Queen and by the children of the Duke and Duchess with other relatives who will go down from town on Thursday evening. After a last night spent on board the vessel they will journey up to Victoria on Saturday, reaching London about one o'clock. The drive to Marlborough House *via* Grosvenor Place, Piccadilly, and St. James's Street, will partake somewhat of a processional character, and there is no doubt that thousands of Londoners will line the streets and give a vociferous welcome to the home-comers. In addition to the pleasure of having their son and daughter-in-law near them again the King and Queen will be particularly glad that in her present delicate state of health Princess May should be able to take things more quietly, and not be always attending functions and festivities. At the reception at Government House, St. John's, Newfoundland, last week, the Duchess, though naturally very strong, was taken suddenly ill, and the ceremony in consequence had to be much curtailed, showing that the continuous strain had told upon her. The indisposition was, however, evidently only of a temporary nature, for she was well enough in the evening to attend a banquet.

It has been often said that no occupation is so wearing, or so apt to produce overwrought nerves, as continuously teaching the young idea to shoot. But evidently her profession has had no bad effect upon the American lady, Mrs. Anna Edson Taylor, who has recently "shot" Niagara. She is a widow, 43 years of age, and earns her livelihood by teaching. Her object in undertaking so dangerous a feat was to raise sufficient money to pay off a mortgage on her western ranch. It does not appear if the money was to be paid just the same, whether she was dead or alive, at the end of her journey; because, if not, it is difficult to see the advantage which would have accrued to the estate if the owner had killed herself, but as she came triumphantly through the rather foolish ordeal, that is not a point of much moment. No human being before has ever made a similar trip and survived to tell the tale. The lady was packed into a barrel 6 feet long, the bottom smaller that the top. Heavy weights at the bottom held the barrel in an upright position. Inside straps were placed over the woman's shoulders to prevent her head from striking the top. The head was also protected by a pillow which she clasped with her hands. After the "shooting" it was fully two minutes after the barrel had been lost to view in the mist and spray at the bottom of the waterfall before it was seen again, which must have been an anxious period for the onlookers. It was promptly hauled ashore, the top sawn off, and instead of finding inside a half dead woman, a bright voice exclaimed, "Have I really gone over the Falls?" The barrel was one-third full of water, which had probably entered through the ventilation holes, and there was a slight contusion on the incarcerated lady's head. Otherwise all was in order. It is to be hoped that the apparent ease with which the feat has been accomplished will not result in other women, who have no mortgages to pay off, following Mrs. Taylor's example.

Some months ago, as you may remember, a Scotch lady endeavoured to follow the example of her French sisters and applied for permission to qualify as a lady lawyer. After considerable discussion, which at first seemed favourable to the applicant, her request was refused. Now an Irish lady, daughter of Sir John B. Johnston, of Londonderry, has made a similar application, her educational record being especially good. The subject was debated at a private meeting of the Benchers in Dublin last week, and the question was put as to whether the lady should be admitted as a student at King's Inn or not. She had several supporters among the Benchers, but the majority were against the admission, and the application was not granted. Although there is no doubt that ultimately all opposition will be withdrawn, the time is not yet.

The wretched man who killed President McKinley has paid the penalty of his outrage, and was executed by electricity early on Tuesday morning. He is the fifty-ninth murderer who has suffered capital punishment in this manner in the State of New York. Up to the last Czolgosz remained stolid and indifferent, slept well nearly all the night through till five A.M., when, although he slept no more, he lay quietly down upon his bed and waited till six. Then he dressed and partook of a hearty breakfast. Then the inordinate vanity and love of notoriety which always seem characteristics of those who commit these repulsive crimes broke out, and the prisoner made a request that he might make a statement where there were "lots of people." On being told that he could not do this, he said, "then I won't talk at all," and with the exception of a brief speech in the chair, saying that he was not sorry for what he had done, and expressing regret that he had not seen his father, he kept his word. He had previously told the priests that he would not re-embrace the Roman Catholic religion, nor renounce anarchism. Though the prison authorities would probably have declined their services, the Buffalo Cremation Company have relieved their feelings of disgust by declining to cremate the remains of Czolgosz, but a woman Socialist of Chicago is endeavouring to raise a large sum of money, £8,000 if possible, to build a mausoleum in his name. It is not surprising that even the Anarchists and Socialists refuse to devote their money in immortalising one special member of their community.

Some of you may like to know that the Committee on Social Education of the Charity Organisation Society have arranged for a course of six lectures, intended for those interested in social subjects, to be given at the Royal United Service Institution, on Fridays at 4.30 P.M., beginning on November 1st. The first three lectures will be delivered by Mr. C. C. J. Webb, Fellow of Magdalen College, Oxford, on "Social Ideals in their Relation to Social Life," and the last three by Mr. C. S. Lock, Secretary to the Charity Organisation Society, on the "Theory and Method of Personal Charity." Particulars may be obtained from the Hon. Sec., Mrs. G. F. Hill, 10 Kensington Mansions, S.W.

### Everybody's Opinion.

[Correspondence on all subjects is invited, but we cannot in any way be responsible for the opinions expressed by our correspondents. No communication can be entertained if the name and address of the correspondent is not given, as a guarantee of good faith but not necessarily for publication, or unless one side of the paper only is written on.]

### A QUESTION OF PRINCIPLE AT BRISTOL INFIRMARY.

"Robert H. Norgate," resident medical officer at Stapleton Workhouse, Bristol, writes: The statements made in the communication forwarded by a correspondent "whose knowledge should be unimpeachable," is quite incorrect so far as it asserts that a certificate of training is to be given to an untrained nurse at the Stapleton Workhouse. It is a fact that there is an excellent nurse employed here, who has been in the sick-ward some five years, and is in every way capable and deserving, but no certificate of training has ever been promised by me, or by the guardians, to her. I have never given such a certificate to any nurse who has not completed the usual period of training. The guardians would not favour such a practice.

### THE SCARCITY OF NURSES IN WORKHOUSE INFIRMARIES.

"A Workhouse Nurse" writes: I have taken a keen interest in your remarks about the scarcity of workhouse nursing. I myself held the post of head nurse in a small country union. Here we have not experienced any difficulty in getting nurses but in keeping them. What, however, can guardians expect if they allow untrained uneducated matrons to interfere with trained nurses' duties? My opinion is that unless the guardians raise the standard of workhouse matrons—I do not mean trained matrons, because a trained matron always behaves like a lady to her nurses—they will always have a difficulty in getting trained nurses. I am leaving my present post, and before deciding to take another in a workhouse I should certainly ask the guardians if the matron was trained, and, if not, what authority she was allowed to have over the nurses. I have two assistant nurses, both very competent women, but I am sorry to say that they have been compelled to resign their posts for the same reason as myself—they object to be insulted by an untrained woman.

### THE NURSING OF OPHTHALMIA NEONATORUM.

"H. H.," a certificated ophthalmic nurse, writes: I think it might interest a few of your readers if I were to state what is required from a nurse attending to babies with this very dreadful disease. Often I have seen cases brought to eye hospitals when it was almost too late to expect any good result, owing either to the carelessness or neglect of the monthly nurse, and to me it seems most sad that so very many cases of total blindness occur simply through the want of knowledge of those attending upon the birth of an infant. Of course, in many cases it is ignorance which is to blame. But it seems terribly hard that ignorance in our enlightened age should cause the blindness of one of God's creatures. Often I have been in hospital and seen babies brought ten days or two weeks old, with the sight completely lost in both eyes, and if only they had been brought directly the disease started they could have been cured. The disease being very infectious great care must be taken by the nurse that everything used must be carbolised after using and kept separate for the use of that one case. Also, she must thoroughly carbolise her hands after attending to the baby; in fact no one can be too careful about washing and carbolising the hands after attending to a patient with ophthalmia, as the disease is most contagious. The easiest way for the nurse to do the baby's eyes is for her to sit on a chair and put over her knees a mackintosh apron, the person holding the baby to sit opposite, and place the baby's head between the knees of the nurse, so that the nurse is then able to hold the patient's head tightly between her knees while the attendant holds on her knee the baby's body. In this way the nurse is enabled to have both her hands free to attend to the baby's eyes. As a rule the swabbing with nitrate of silver is continued till the pus has stopped. But, of course, the nurse will be told by the surgeon. Still, a great deal must depend upon the nurse, for if she is careless or rough the eye will perforate and the lens come out. She must also be very careful that when separating the lids of the patient the discharge does not spurt up into her own eyes. If by any chance this was to happen she must at once get her eye washed out with 1-5,000 sublimate, and let the doctor see her eyes as soon as possible.

### WHY ARE NIGHT NURSES NEGLECTED?

"A Sympathiser" writes: Can you tell me why night nurses are so disregarded, seeing that they are such a very necessary and important article? With the majority of patients who are really ill the night is the most trying time and the time when they need the most care and attention. Yet in some institutions that I know of and have worked in the night nurses are quite disregarded; even the maids seem to think anything is good enough for them. The tables are laid carelessly, the meals are not so punctual as they are for the day staff. The food is badly cooked and not served up as tastefully as for the day people. Sometimes what has been ordered for the table is not put on to it. The maids or the cook do not trouble to remember or notice what has been ordered, and just produce what causes them the least trouble, because "it is only for the night nurses." Is it because the matron does not preside at their table? But all round there seems to be an air of carelessness about the treatment of the night nurses. Even if there has been decent food ordered for the night they seldom get it. Often untemptingly cut cold meat with an allowance of tea and bread and a minute proportion of butter is all that is provided, and they have to rely on being able to get milk from the patients' allowance for their tea. They rarely see jam and other things that are provided for the day staff, or if so in very small proportion. For their final meal when coming off duty in the morning, feeling tired, after sometimes a heavy and trying night of 12 hours, the food is anything but appetising, and all that is usually provided to drink is cold water. In some institutions tea or coffee is provided, but it is often made so badly that it is not fit to drink. I have known night nurses spend the greater part of their salary in buying food to prevent their health giving way. Seeing that they have the most responsible and trying time, and sometimes at night when the wards are heavy are unable to snatch even 10 minutes for refreshment, ought they not to be considered more than they are? Their sleeping quarters cannot always be arranged in a secluded part, and the maids and people about in the day give no thought to the tired nurses who have been on duty all night and are trying to rest for the coming night, when others will be comfortable in their beds. Even when the work is lighter—as it often varies—there is always a great nervous strain, for more deaths and changes in illness occur in the night than in the daytime. The nursing subject seems to be creating general interest at the present day. Ought not the toilers of the night to be more considered than they are? Even their holidays are curtailed. Day nurses in many institutions now have one or two half-days besides one day a month allowed them, but the poor night nurses have to be contented, and sometimes grateful, too, if they can get one night off during the month.

### LIFE IN A NAVVY HOSPITAL.

"Geraldine Hamilton" writes from Tyla Morris, Pentyrch, near Cardiff: While reading with much interest the kindly and sympathetic account of "Life in a Navvy

Hospital" in your paper of September 28th I could not help being struck by the fact that the writer has, I am sure quite unintentionally, given fresh currency to a word-portrait of navvies as a class, which, while unfortunately too true of some of these men, is quite a libel upon a very large and increasing number of them. I refer to the passage describing navvies thus: "No change of dress Sunday or week-days; always the public-house when off duty." And again: "They [the navvies] have . . . their own craving for strong spirits and power of satisfying it. They earn good wages, but spend nearly all in drink." I have learnt from intimate acquaintance and friendship, for several years, with large bodies of navvies within a radius of 15 miles from my home, how acutely sensitive the many to whom these words do not apply, are to the evil reputation which they do undeservedly bear; they will tell you almost with tears, "We went (in such good Sunday clothes, poor fellows!) to such a place of worship, but they could see we was navvies;" or of how a newspaper said that "four men and two navvies had been killed somewhere, and how a photographer, seeing two of them wearing Christian Union badges, had exclaimed, 'Dear me, I didn't know there were any Christians amongst navvies!'" On the other hand, I was told quite spontaneously by a shopkeeper a year ago that a certain street in a large town had "so improved since the navvies came, they are nice people?" and a month ago I heard a clergyman make the same remark about a whole district in that town. It would, I think, be a real benefit, both to the navvies and to the inhabitants of the districts where they settle, if the public could learn once for all that, whatever may have been the case in bygone years, at the present time the advent of a body of navvies into a neighbourhood does not mean that a horde of disorderly drunkards are coming to invest the place and cause disturbances, but that in very many instances, accompanied as they are by one of the Navvy Mission Society's missionaries (the best of whom have been navvies themselves) they bring with them a great increase of spiritual life and activity, and many noble examples of earnest and unselfish lives, dedicated, amidst difficult surroundings, to the service of God and their fellows. At a seaport town near here I was at a harvest thanksgiving service in the Navvy Mission-room on September 29th. It is now more than a year since the missionary was withdrawn from this station, owing to pressing needs elsewhere; but the band of "Christian Excavators' Union" members who had gathered round during his five years' ministry (all navvies) are carrying on the whole work of the Mission since he left, taking Bible classes, Sunday schools and Sunday services. We found a congregation of 350 people in the mission room, which was profusely—lavishly—decorated with flowers, fruit and vegetables, all given by the people, to be "bought back" next day for the benefit of the Sunday schools, etc. I think it would have done your correspondent's heart good to see the Sunday clothes and the Sunday faces, and hear the hearty singing and earnest, eloquent prayers of these dear people, who should, in common justice be set free from the "bad name" which has too long been flung at them. I could multiply instances to prove what I have stated, but fear I have already trespassed too much on your space.

### To Nurses.

We invite contributions from any of our readers, and shall be glad to pay for "Notes on News from the Nursing World," or for articles describing nursing experiences, or dealing with any nursing question from an original point of view. The minimum payment for contributions is 5s., but we welcome interesting contributions of a column, or a page, in length. It may be added that notices of entertainments, presentations, and deaths are not paid for, but, of course, we are always glad to receive them. All rejected manuscripts are returned in due course, and all payments for manuscripts used are made as early as possible after the beginning of each quarter.

### Maternity Charity and District Nurses' Home at Plaistow.

### OPENING OF THE NEW BUILDING.

On Tuesday afternoon the new rooms built for the Nurses' Home, at Howard's Road, Plaistow, were inspected by friends and subscribers, and a reception was held by the Mayor and Corporation, the Mayor wearing his chain of office, and being accompanied by the Mayoress. The reception took place in the nurses' new dining-room, where short speeches were made on the objects and financial position of the charity.

After some introductory remarks by the Mayor, Mr. James, the organising secretary, gave a brief account of the home, and announced that a bazaar would take place in Stratford Town Hall on November 25th and 26th, in aid of the funds.

Mr. Alderman Adamson said that the charity was greatly indebted to the late Sir Henry Tate, but for whose generous benefaction the nurses would have had to "muddle on" as they had done before, in an inadequate house. One or two rooms still remained to be furnished.

The Bishop of Colchester observed that the last time he met the mayor it was at an athletic meeting where bones were liable to be broken; this, on the contrary, was a meeting of an association that undertook to mend broken bones. He hoped the corporation of West Ham were prepared to do their duty by the nurses; they had already passed them a vote of thanks for their invaluable help during the epidemic of typhoid, and he thought that they ought to feel prepared to pay for the help they so much appreciated. Eighty nurses could now be accommodated in the building, and work could be found for yet a larger number. It was a great joy to him to know that the nurses, whose labours were so arduous, had a comfortable home, for unless they were well fed and well slept they could not do their work. Since he first heard of this work he had felt an interest in it, for he saw how valuable it was all round. There was hardly a county in England that had not a nurse trained by Sister Katherine. Nurses were trained to work in cottages, where they had to make shift with what appliances they could get and where people were often feckless and incapable; for such work this training was invaluable. The nurses in Plaistow and West Ham brought untold blessings into the homes of the poor, and they were able to render splendid services to the labourers at the docks, where accidents frequently happened. Moreover, it was a Christian institution, and he would always say that the nurse came next to the parson; indeed, he doubted whether she was not sometimes of more value than the parson. They were being sent out through the kingdom to take the place of Mother Gamp. He wished the vote of thanks had been offered to Sister Katherine instead of to the Corporation, for though the latter deserved it, he considered that the Borough of West Ham owed a great deal to the institution.

Two of the doctors under whom the nurses work having borne testimony to the value of their work in the district, the Mayor said that the thanks of the borough were due to Sister Katherine rather than to anyone else. He and the doctor and town clerk looked upon the engagement with the nurses as a financial one; they fully recognised that they would have to pay, and the matter would be gone into. He was well aware that the Corporation's vote of thanks could not pay for all the nurses had done during the epidemic.

The Bishop of Barking having arrived, a few words were demanded from him; he alluded to the help Sister Katherine's nurses had given on the occasion of the launching of a ship, when a terrible accident had happened, and the injured were tended by the nurses, who arrived almost instantly on the scene.

After the speeches the visitors inspected the rooms; these provide accommodation for thirty additional nurses, including bedrooms, day and lecture rooms, a set of apartments for sick nurses, eight bathrooms, storerooms, etc. The entire building is painted a light buff, and there is no attempt at decoration, except in the choice of furniture, the general effect being plain and clean. Most of the nurses were present at the opening ceremony.

### For Reading to the Sick.

ALL SAINTS' DAY.

"*The memory of the just is blessed.*"—Prov. x. 7.

THEY rest from all their labours,
Yet serve Him day and night;
Their earthly forms are sleeping
But they, in deep delight,
Wait for the Resurrection,
Of Life the perfect Crown,
The time of Restitution,
Christ's triumph, and their own.
O think of that assembly!
Their beauty and their peace;
Souls perfect, yet receiving
Love's infinite increase.
In full illumination,
Knowing as they are known,
The transitory ended
And the imperfect flown. *C. N. Noel.*

They who love in the Lord never see each other for the ast time.—*Anon.*

The Commemoration of Saints is one of the provisions which has been wisely made by our Church to bring home to us our connection with the invisible life; to help us to confess that they who once lived to God live still; to know that we are heirs not of a dead past, but of a past fresh with new lessons; to learn that consecrated gifts become an eternal blessing; to understand — most touching mystery — that Christ is pleased to reveal Himself little by little, "in many parts and in many fashions," in the persons of His servants. Thus it is that each saint receives and shows some trait of the perfect Manhood of his Master. And "We that are but parts" can recognise in a scale suited to our weakness, now this grace and now that, according to our needs. Thus it is that slowly and through manifold energies the members show us the grandeur and beauty of the One life by which they are inspired: that we come to feel that there is a place for us also in the vast Temple which is reared through the ages on the Foundation of Christ for the glory of God. . . .

I trust that we call up in grateful memory saints whom we have known—the glory of their devoted service—to give distinctness to thanksgiving and hymn. There is not one among us whose study and whose experience may not bring some dear companion, whom he has learnt to recognise in the silent converse of books or in the stirring conflicts of duty, to swell "the glorious company of the Apostles," and "the goodly fellowship of the prophets," and "the noble army of martyrs," men who in these later days and in our own Church have heard a call of God and have obeyed it, men who have seen a truth of God and have interpreted it, men who have received a burden at the hands of God and in trust on Him have borne it, saints who have not been reckoned in any calendar.—*Bishop Westcott.*

"Who are in God's hand, and round about them thrown
The light invisible of a land unknown;
Who are in God's hand; in quietness can wait
Age, pain, and death, and all that men call Fate:—
What matter if thou hold thy loved ones prest
Still with close arms upon thy yearning breast,
Or with purged eyes behold them hand in hand
Come in a vision from that lovely land,—
Or only with great heart and spirit sure
Deserve them and await them and endure;
Knowing well, no shocks that fall, no years that flee,
Can sunder God from these, or God from thee;
No wise so far thy love from theirs can roam,
As past the mansions of His endless home."
*F. W. H. Myers.*

### Notes and Queries.

**The Editor is always willing to answer in this column, without any fee, all reasonable questions, as soon as possible.**
**But the following rules must be carefully observed:—**
**1. Every communication must be accompanied by the name and address of the writer.**
**2. The question must always bear upon nursing, directly or indirectly.**
**If an answer is required by letter a fee of half-a-crown must be enclosed with the note containing the inquiry.**

*South African Constabulary Nursing Establishment.*

(37) I see in THE HOSPITAL that candidates for the South African Constabulary Nursing Establishment must apply for particulars, etc., to Colonel Beevor in Johannesburg. This is the first announcement I have seen concerning this nursing establishment, and should be glad if you will tell me if I can get any information concerning it nearer home, as it seems such a long way to apply for preliminary information, and also such a long time to wait before any correspondence could be exchanged.—*South Africa.*

All applications must be made as directed to Colonel Beevor.

*Maternity Nursing.*

(38) Will you kindly tell me where I can obtain obstetric lectures in Wimbledon or Croydon?—*S. M.*

Write up to the secretary of the Midwives' Institute, 12 Buckingham Street, Strand, W.C.

I am anxious to learn midwifery in about two years' time. Will you tell me what books I could study meanwhile?—*M. F.*

"A Practical Handbook on Midwifery," by Francis W. N. Haultain, M.D., price 6s., is much recommended. We should advise you to study general nursing as a necessary adjunct to midwifery.

Will you kindly tell me where a trained nurse can get midwifery training without paying fees, or at reduced fees?—*E. H. L.*

The matron, the Maternity Nursing Mission, 171A King's Cross Road, London, W.C., might help you. See our advertisements.

Can you tell me of a lying-in hospital where a patient can be received on payment of 10s. or 13s. a week?—*L. H.*

The Clapham Maternity Hospital, 41 and 43 Jeffrey's Road, Clapham, S.W. Payment 7s. 6d. to 30s. per week, according to circumstances.

Can you tell me of a large town in England where a trained maternity nurse could work up a good private connection?—*M. N.*

This is a point on which we can give no information.

*Floor Stain.*

(39) Kindly let me know how I can stain a ward floor cheaply?—*L. P.*

Permanganate of potash mixed with water until the stain gives the desired depth of colour, answers very well. Polish the floor afterwards with beeswax and turpentine.

*South Africa.*

(40) Will you kindly tell me where I ought to apply for information with regard to being sent out to South Africa?—*K. G. A.*

Apply the Hon. Secretary, the Army Nursing Reserve, 18 Victoria Street, S.W.

*Private Home.*

(41) I am thinking of taking one or two patients, or nurses requiring rest, into my own home. Should I be allowed to do this if my landlord did not object?—*M. T.*

Yes; provided that your neighbours make no objection as to the nature of the complaints from which your patients are suffering and that they are not insane.

*Deafness.*

(42) Would you advise a nurse who is getting deaf to try the —— system? She must work for a living and is much attached to her profession. Could she learn lip-reading? Failing nursing, is there anything else she could do?—*S. F. K.*

Don't tamper with quack nostrums, and don't lose hope until you have consulted a specialist. Advertise for work stating your deafness. Perhaps some of the institutes for the deaf and dumb might utilise your skill in nursing.

**Standard Books of Reference.**

"The Nursing Profession: How and Where to Train." 2s. net; post free 2s. 4d.
"Burdett's Official Nursing Directory." 3s. net; post free, 3s. 4d.
"Burdett's Hospitals and Charities." 5s.
"The Nurses' Dictionary of Medical Terms." 2s.
"Burdett's Series of Nursing Text-Books." 1s. each.
"A Handbook for Nurses." (Illustrated). 5s.
"Nursing: Its Theory and Practice." New Edition. 3s. 6d.
"Helps in Sickness and to Health." Fifteenth Thousand. 5s.
"The Physiological Feeding of Infants." 1s.
"The Physiological Nursery Chart." 1s.; post free, 1s. 3d.
"Hospital Expenditure: The Commissariat." 2s. 6d.

All these are published by the SCIENTIFIC PRESS, Ltd., and may be obtained through any bookseller or direct from the publishers 28 and 29 Southampton Street, London, W.C.

## Figures and Tables

**Fig. 4. Fig. 5. Fig. 6. f1:**
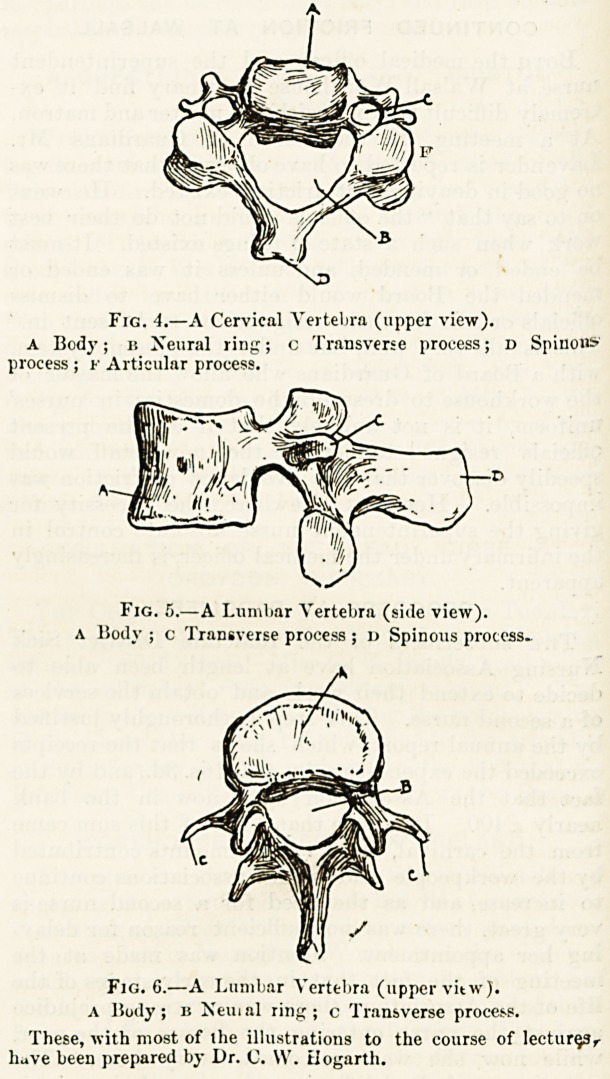


**Figure f2:**